# Pharmacodynamic assessment of prasugrel and clopidogrel in patients with non-cardioembolic stroke: a multicenter, randomized, active-control clinical trial

**DOI:** 10.1007/s11239-019-01926-6

**Published:** 2019-10-23

**Authors:** Takenori Yamaguchi, Toshiaki Shirai, Satoshi Yoshiba, Kenji Abe, Yasuo Ikeda

**Affiliations:** 1 National Cerebral and Cardiovascular Center, 5-7-1 Fujishiro-dai, Suita, Osaka 565-8565 Japan; 2 Daiichi Sankyo Co., Ltd., 1-2-58 Hiromachi, Shinagawa-ku, Tokyo, 140-8710 Japan; 3 Faculty of Science and Engineering, Waseda University, 3-4-1 Okubo, Shinjuku-ku, Tokyo, 169-8555 Japan

**Keywords:** CYP2C19, Inhibition of platelet aggregation, Platelet aggregation, Platelet reactivity index, Vasodilator-stimulated phosphoprotein

## Abstract

Prasugrel, a novel P2Y_12_ receptor antagonist, has been shown to be more effective than clopidogrel for preventing cardiovascular events in patients with acute coronary syndromes undergoing percutaneous coronary intervention. We investigated the dose–response antiplatelet effects of prasugrel compared with clopidogrel in Japanese patients with non-cardioembolic stroke. The influence of cytochrome P450 (CYP) polymorphisms on the antiplatelet effects of both drugs was also compared. In this multicenter randomized active-control comparative study, patients were randomized to receive prasugrel 2.5 mg, 5 mg, or 7.5 mg (double blind) or clopidogrel 75 mg (open label) once daily for 14 days. The primary endpoint was inhibition of platelet aggregation (IPA) in response to adenosine diphosphate 20 μM within 8 h of study drug administration on day 14. Of the 66 patients randomized, data from 63 (prasugrel 2.5 mg, 5 mg, and 7.5 mg groups, *n* = 14, 16, and 18, respectively; clopidogrel group, *n* = 15) were used in the pharmacodynamic assessment. IPA (arithmetic mean ± SD) after prasugrel administration increased dose-dependently (33 ± 9%, 44 ± 11%, and 53 ± 14%, at 2.5 mg, 5 mg, and 7.5 mg, respectively) and was higher in these groups than after clopidogrel (23 ± 16%). In a subgroup of CYP2C19 intermediate metabolizers, IPA was higher in the prasugrel 5 mg and 7.5 mg groups than in the clopidogrel group. No death or serious adverse events were reported. Prasugrel was well tolerated at doses up to 7.5 mg/day and had antiplatelet effects higher than those of clopidogrel 75 mg/day. CYP2C19 polymorphisms may have reduced clopidogrel-induced IPA.

**Electronic supplementary material:**

The online version of this article (10.1007/s11239-019-01926-6) contains supplementary material, which is available to authorized users.

## **Highlights**

- Antiplatelet drugs aspirin, cilostazol, and clopidogrel are recommended in the most recent Japanese guidelines for stroke management, however, poor or non-response to clopidogrel and aspirin have been reported.
- Clopidogrel resistance is reported to be associated with CYP2C19 polymorphism.
- As poor or non-response to antiplatelet drugs presents a risk for another stroke in ischemic stroke patients, the use of the alternative antiplatelet drug, prasugrel, which selectively inhibits the P2Y12 subtype of adenosine diphosphate receptors, is gaining attention.
- We investigated the dose-response antiplatelet effects of prasugrel (2.5 mg, 5 mg, or 7.5 mg) compared with clopidogrel (75 mg) in Japanese patients with non-cardioembolic stroke and found that prasugrel showed higher antiplatelet effects for all three doses than clopidogrel.
- In addition, our findings suggest that CYP2C19 polymorphisms may have reduced clopidogrel-induced inhibition of platelet activation, whereas prasugrel was unaffected.

## Introduction

Despite the use of the antiplatelet drugs aspirin, cilostazol, and clopidogrel, as recommended by the most recent Japanese guidelines for the management of stroke [1], survivors of ischemic stroke remain at risk of another stroke. Clopidogrel, which is widely used for stroke prevention, is a prodrug whose antiplatelet effects depend on conversion into its active metabolite by enzymes including cytochrome P450.

Poor or non-response to clopidogrel has been reported in a significant proportion of patients. This phenomenon, known as clopidogrel resistance, is associated with genetic polymorphisms; the presence of variants of the gene for the cytochrome P450 subtype CYP2C19, in particular, limits metabolic activation of clopidogrel and thus reduces clopidogrel-induced inhibition of platelet activation (IPA) [2–9]. Patients treated with clopidogrel who carry one or two reduced-function *CYP2C19* alleles have been shown to be at increased risk of major adverse cardiovascular events [10, 11]. Furthermore, reduction of clopidogrel-induced IPA due to CYP2C19 polymorphisms has been reported in patients with ischemic stroke [12]. Therefore, *CYP2C19* genotype may need to be considered when choosing the antiplatelet drug most likely to be effective in individual patients.

Prasugrel has been developed as a third-generation thienopyridine antiplatelet drug. Like clopidogrel, prasugrel is a prodrug that exerts its antiplatelet effects, via an active metabolite, by selective inhibition of the P2Y_12_ subtype of the adenosine diphosphate (ADP) receptor. However, compared with clopidogrel, prasugrel is a more potent and faster-acting inhibitor of platelet aggregation [13]. Furthermore, the antiplatelet effects of prasugrel are more consistent. With prasugrel, exposure to the active metabolite and pharmacodynamic response are unaffected by CYP2C19 and CYP2C9 polymorphisms; in contrast, with clopidogrel they are reduced [14].

The results of an international, large-scale randomized clinical trial in patients with acute coronary syndromes has shown prasugrel to be more effective than clopidogrel in the prevention of cerebro- and cardiovascular events [15]. Therefore, prasugrel is expected to be similarly effective in patients with non-cardioembolic stroke.

Our main aims in carrying out the present study were to investigate the dose–response antiplatelet effects of prasugrel and to compare the antiplatelet effects of prasugrel and clopidogrel in patients with non-cardioembolic stroke. We also investigated the influence of CYP2C19 polymorphisms on the antiplatelet effects of both drugs.

## Methods

### Study population

Patients were eligible for the study if they were aged 20–74 years and had had a non-cardioembolic stroke at least 4 weeks previously. The exclusion criteria were modified Rankin Scale score ≥ 4 or DSM-III-R severe or above; cardioembolic stroke; cardiovascular disease with the potential risk to cause cardioembolic stroke; unruptured intracranial aneurysm ≥ 5 mm; high bleeding risk; uncontrolled hypertension; uncontrolled diabetes; liver dysfunction, severe blood disorder, and severe renal dysfunction, as per the criteria defined in the protocol; heart failure of New York Heart Association class III or IV, or severe arrhythmia; and maximum platelet aggregation (MPA) in response to ADP 20 μM < 50%.

### Study design

This was a multicenter, randomized, active-control study carried out at seven hospitals in Japan. Patients were randomly allocated in a 1:1:1:1 ratio to receive prasugrel 2.5 mg, 5 mg, or 7.5 mg (by double-blind administration) or clopidogrel 75 mg (by open-label administration) once daily for 14 days. This treatment period was preceded by a pretreatment period of ≤ 30 days and followed by a follow-up period of 14 days (Supplemental Fig. 1). Blood samples were collected during the pretreatment period and within 8 h of administration of the study drug on day 14 (i.e. the last day of the treatment period).

The study was conducted in accordance with the Declaration of Helsinki, Japanese Pharmaceutical Affairs Law, and Good Clinical Practice. The study protocol was approved by the institutional review board at each hospital, and written informed consent was obtained from all participants.

### Pharmacodynamic assessment

The primary endpoint of the study was IPA in response to ADP 20 μM within 8 h of study drug administration on day 14. Secondary endpoints were IPA at other blood-sampling time points and platelet reactivity index (PRI) at each time point.

Inhibition of platelet aggregation in response to ADP 20 μM (CHRONO-PAR ADP reagent; Chrono-log Corporation, Havertown, Pennsylvania, USA) was determined by light transmission aggregometry, as follows. At each blood-sampling time point, MPA was measured using a platelet aggregation analyzer (MCM Hematracer 313 M; MC Medical Inc., Tokyo, Japan). The MPA values were then used to calculate IPA in response to ADP 20 μM by using the following formula:

$$ {\text{IPA}}\; \left( \% \right)\; = \;\frac{{{\text{Pretreatment}}\;{\text{MPA}} - {\text{MPA}}\;{\text{at}}\;{\text{each}}\;{\text{blood}}\;{\text{sampling}}}}{{{\text{Pretreatment}}\;{\text{MPA}}}}  \times 100 $$

Platelet reactivity index was determined by flow cytometric analysis of the phosphorylation state of vasodilator-stimulated phosphoprotein (VASP). It was calculated at each blood-sampling time point by using the following formula:

$$ {\text{PRI}} =  \frac{{{\text{MFI}}_{{{\text{PGE}}1}} - {\text{MFI}}_{{{\text{ADP}}}} }}{{{\text{MFI}}_{{{\text{PGE}}1}} }}  \times 100 $$

$$ {\text{MFI}}_{{{\text{PGE}}1}} =  {\text{MFI}}\;\left( {{\text{T}}1} \right)\;{-}\;{\text{MFI}}\;\left( {{\text{T}}3} \right) $$

$$ {\text{MFI}}_{{{\text{ADP}}}} =  {\text{MFI}}\;\left( {{\text{T}}2} \right) {-}{\text{ MFI}}\;\left( {{\text{T}}3} \right). $$

In the formula, MFI stands for mean fluorescence intensity; PGE1, prostaglandin E1; MFI (T1), MFI after exposure of the sample to PGE1, when stained with anti-phosphorylated VASP antibodies; MFI (T2), MFI after exposure of the sample to ADP and PGE1, when stained with anti-phosphorylated VASP antibodies; and MFI (T3), MFI after exposure of the sample to ADP and PGE1, when stained with negative control antibodies.

### Pharmacokinetic assessment

To measure plasma concentration of the active metabolites of prasugrel and clopidogrel (R-138727 and R-130964, respectively), blood samples were collected on day 14, within 8 h of administration of each drug. At each blood-sampling time point, 5 mL of venous blood was collected by vacuum extraction into a blood-sampling tube containing EDTA sodium. Immediately after collection, 25 μL of 3′-methoxyphenacyl bromide–acetonitrile solution (0.5 M) was added to the sample and the resulting solution mixed by inverting the tube before placing it on ice to chill.

Plasma obtained by centrifugation (4 °C, 3000 rpm, 10 min) was transferred into storage containers and stored frozen (below − 20 °C). The plasma concentration of the active metabolites of prasugrel and clopidogrel was measured by liquid chromatography–tandem mass spectrometry.

### Safety assessment

Safety assessment included incidence of adverse events (AEs), changes in laboratory values, and vital signs. AEs reported as bleeding events were defined as follows: *major bleeding*, life-threatening bleeding or intracranial bleeding; *clinically relevant non-major bleeding*, bleeding not defined as major bleeding but requiring discontinuation of study treatment at the discretion of the investigators; *other bleeding*, bleeding events not defined as major bleeding or clinically relevant non-major bleeding.

### Genotyping

*CYP2C19* genotype was determined by analysis of genomic DNA obtained from blood samples. Patients were then classified into three phenotype groups accordingly: extensive metabolizers (EMs), with no mutant alleles; intermediate metabolizers (IMs), with one mutant allele; and poor metabolizers (PMs), with two or more mutant alleles. The data were used to investigate relations between CYP2C19 phenotype and IPA, PRI, and plasma concentration of the active metabolite of each study drug.

### Statistical analysis

To assess the relation between prasugrel dose and IPA that is the main objective of the study, the target number of patients in each prasugrel group was established as follows. Based on the results of the Japanese phase I multiple-dose study [16], IPA (mean ± SD) in response to ADP 20 μM was assumed to be 20.0 ± 25%, 58.9 ± 20%, and 68.3 ± 15% in the 2.5 mg, 5 mg, and 7.5 mg groups, respectively. These values were used in a simulation with 1000 repetitions and 14 patients in each treatment group, which showed a 91.1% probability of monotonicity of arithmetic means and a ≥ 90% probability of a dose–response relation. The simulation also showed that if the number of patients in each treatment group were reduced to 12 (e.g. due to dropouts), the probability of monotonicity of arithmetic means would be 88.6%. Therefore, the target number of patients in each prasugrel group was set at 14. The same target number was set for the clopidogrel group to enable comparison at the same level of precision.

Unless otherwise specified, data from the patients in the per-protocol set were used for the pharmacodynamic and pharmacokinetic analyses. The safety analysis set comprised all patients who had received at least one dose of either of the study drugs.

For the pharmacodynamic analyses, summary statistics for MPA in response to ADP 20 μM on day 14 were generated for each treatment group. These values were then used to calculate IPA for each patient; summary statistics were generated for each treatment group at each blood-sampling time point. The relation between prasugrel dose and IPA was investigated by regression analysis using a linear model.

The results of the flow cytometric VASP assay were used to generate summary statistics for PRI for each treatment group at each blood-sampling time point.

For the safety assessment, data on AEs (signs and symptoms as well as abnormal changes in laboratory data) were tabulated.

Statistical tests were carried out using SAS System Release 8.2 (SAS Institute Inc., Cary, North Carolina, USA). The significance level was set at 5%.

## Results

The study was conducted between February 20, 2007 (when the first patients were recruited) and September 19, 2007 (the date of the final patient visit).

### Patient flow and baseline characteristics

**Fig. 1:**
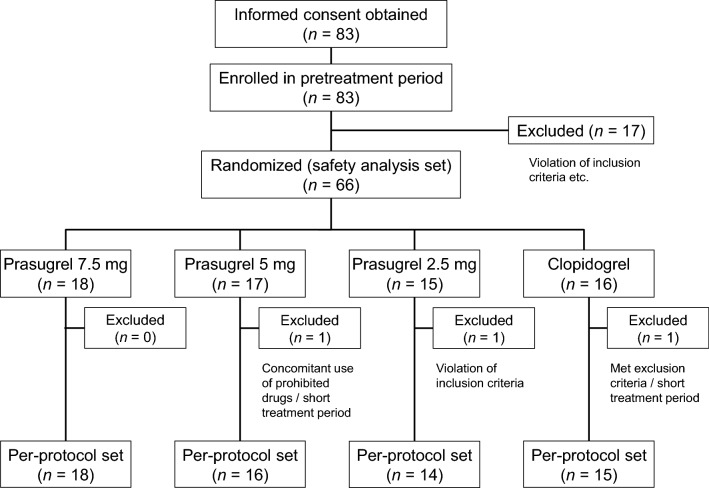
Flow of patients through the trial

Figure 1 shows the flow of patients through the phases of the trial. Of 66 patients randomly allocated to a treatment group, three were excluded for the reasons shown in Fig. 1. Therefore, the per-protocol set comprised 63 patients: 14 in the prasugrel 2.5 mg group, 16 in the prasugrel 5 mg group, 18 in the prasugrel 7.5 mg group, and 15 in the clopidogrel group.

The baseline characteristics of patients in the per-protocol set are summarized in Table 1. The treatment groups were similar in terms of mean age (range 62.3–66.1 years) and body weight (62.1–68.7 kg). The prasugrel 7.5 mg group and the clopidogrel group had a high proportion of male patients. Compared with the clopidogrel group, the prasugrel groups had a slightly higher proportion of patients with lacunar infarction. There were no other noteworthy intergroup differences.

**Table 1:** Baseline characteristics of the patients in the per-protocol set

Characteristic	Treatment group			
	Prasugrel 7.5 mg (*n* = 18)	Prasugrel 5 mg (*n* = 16)	Prasugrel 2.5 mg (*n* = 14)	Clopidogrel 75 mg (*n* = 15)
Age (years)	62.3 ± 9.6	66.1 ± 6.2	63.3 ± 8.7	65.2 ± 9.2
Male, *n* (%)	15 (83.3)	9 (56.3)	9 (64.3)	12 (80.0)
Height (cm)	164.0 ± 7.8	160.0 ± 9.6	158.5 ± 6.5	162.2 ± 6.5
Weight (kg)	68.7 ± 12.2	64.0 ± 11.4	63.5 ± 8.7	62.1 ± 8.1
Body mass index (kg/m^2^)	25.5 ± 3.7	24.9 ± 3.1	25.3 ± 2.6	23.6 ± 2.5
Time between most recent stroke and start of treatment (days)	1769.2 ± 1728.0	2065.8 ± 2095.7	1504.1 ± 1622.0	1421.6 ± 1213.5
Diabetes mellitus, *n* (%)	16 (88.9)	12 (75.0)	11 (78.6)	12 (80.0)
Type of most recent stroke, *n* (%)				
Lacunar infarction	14 (77.8)	13 (81.3)	12 (85.7)	9 (60.0)
Atherothrombotic infarction	3 (16.7)	3 (18.8)	2 (14.3)	6 (40.0)
Unknown	0 (0.0)	0 (0.0)	0 (0.0)	1 (5.6)
Phenotype according to CYP2C19 status^a^, *n* (%)				
Extensive metabolizer	6 (46.2)	7 (50.0)	7 (70.0)	1 (7.7)
Intermediate metabolizer	6 (46.2)	6 (42.9)	1 (10.0)	11 (84.6)
Poor metabolizer	1 (7.7)	1 (7.1)	2 (20.0)	1 (7.7)

Values are expressed as the arithmetic mean ± standard deviation, unless otherwise stated

^a^The denominator is the number of patients whose phenotype was confirmed in each treatment group as follows: prasugrel 7.5 mg (*n* = 13); prasugrel 5 mg (*n* = 14); prasugrel 2.5 mg (*n* = 10); clopidogrel (*n* = 13)

Patients in the per-protocol set and the safety analysis set had similar baseline characteristics (data not shown).

### Pharmacodynamic assessment

#### Inhibition of platelet aggregation

**Fig. 2:**
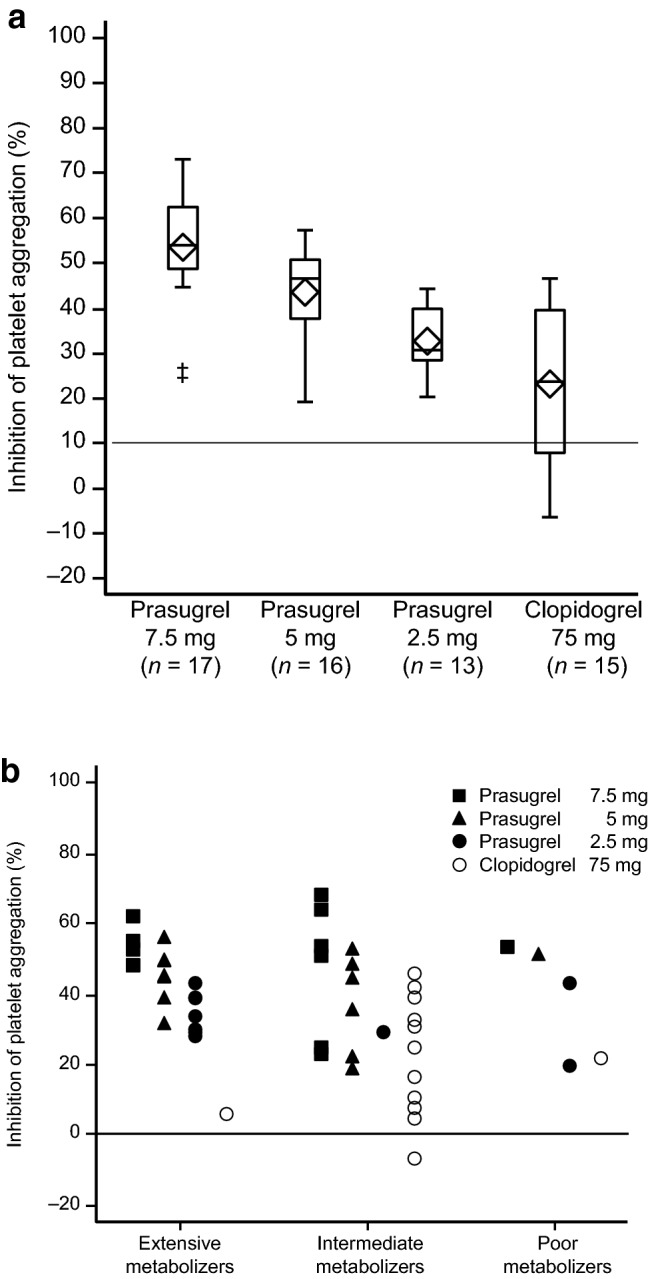
**a** Inhibition of platelet aggregation in response to adenosine diphosphate 20 μM on day 14. Open diamond, arithmetic mean; double dagger, value > 3 quartiles from the box. Data from one patient in the prasugrel 2.5 mg group and one in the prasugrel 7.5 mg group were insufficient (due to hemolysis of blood samples) so have been excluded. **b** Inhibition of platelet aggregation in response to adenosine diphosphate 20 μM on day 14 in patients classified by CYP2C19 phenotype: filled square, prasugrel 7.5 mg group (5 extensive metabolizers [EMs], 6 intermediate metabolizers [IMs], 1 poor metabolizer [PM]); filled triangle, prasugrel 5 mg group (7 EMs, 6 IMs, 1 PM); filled circle, prasugrel 2.5 mg group (7 EMs, 1 IM, 2 PMs); open circle, clopidogrel 75 mg group (1 EM, 11 IMs, 1 PM)

Figure 2a shows the results for the primary endpoint of the study, namely IPA in response to ADP 20 μM on day 14. It shows a dose–response relation between prasugrel dose and IPA; IPA (mean ± SD) increased from 33 ± 9% at 2.5 mg to 44 ± 11% and 53 ± 14% at 5 mg and 7.5 mg, respectively. In the clopidogrel group, IPA on day 14 was 23 ± 16%, which was lower than the values for the prasugrel groups. Also, in the clopidogrel group, there was large variation in IPA between individual patients (range − 6.3 to 46.4%).

Clopidogrel resistance was defined as < 10% inhibition of ADP-induced platelet aggregation, as in a previous study [17]. On day 14, 4 of the 15 patients in the clopidogrel group had IPA < 10%, whereas no patients in the prasugrel 2.5 mg group had IPA < 10%. The results showed a significant increase in the inhibitory effect on platelet aggregation with an increase in the dose, and thus demonstrated a dose–response relation (p < 0.0001).

#### Relation between CYP2C19 phenotype and IPA

Figure 2b shows IPA in response to ADP 20 μM in patients with different metabolizer phenotypes according to CYP2C19 status. The great majority of patients in the clopidogrel group were IMs; only one patient was an EM and one a PM. Therefore, the data are insufficient to enable meaningful interpretation in terms of the relation between CYP2C19 phenotype and IPA in patients in the prasugrel groups versus those in the clopidogrel group.

#### Platelet reactivity index

The results show a dose–response relation between prasugrel dose and PRI on day 14 (Supplemental Fig. 2); PRI (mean ± SD) decreased in a dose-dependent manner in the prasugrel groups (20.2 ± 12.1%, 28.8 ± 12.9%, and 50.7 ± 10.8%, at 7.5 mg, 5 mg, and 2.5 mg respectively), which were numerically lower than that in the clopidogrel group (58.5 ± 15.7).

### Pharmacokinetic assessment

**Fig. 3:**
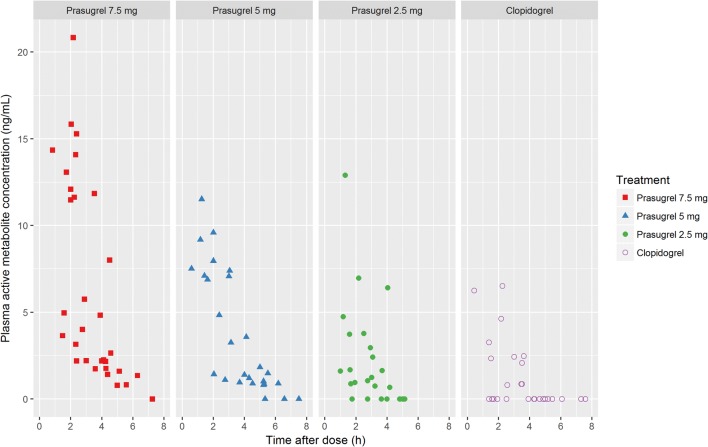
Plasma concentrations of the active metabolites of prasugrel and clopidogrel (R-138727 and R-130964, respectively) in individual patients in each treatment group on day 14

The plasma concentration of the active metabolite of prasugrel tended to be high in all three prasugrel groups, compared with that of the active metabolite of clopidogrel (Fig. 3).

### Safety assessment

Safety data are summarized in Table 2. There were no deaths, other serious AEs (either related or unrelated to the study drug), or discontinuations due to AEs. Bleeding AEs were recorded for 2 of 15 patients in the prasugrel 2.5 mg group, 2 of 17 patients in the prasugrel 5 mg group, 2 of 18 patients in the prasugrel 7.5 mg group, and 1 of 16 patients in the clopidogrel group. All these events were classified as other bleeding; none were classified as major bleeding or clinically relevant non-major bleeding.

**Table 2:** Incidence of adverse events (data from patients in the safety analysis set)

Adverse event	Treatment group			
	Prasugrel 7.5 mg (*n* = 18)	Prasugrel 5 mg (*n* = 17)	Prasugrel 2.5 mg (*n* = 15)	Clopidogrel 75 mg (*n* = 16)
Subjective and objective findings				
All	6 (33.3)	5 (29.4)	3 (20.0)	3 (18.8)
Related to the study drug	1 (5.6)	0 (0.0)	1 (6.7)	0 (0.0)
Abnormal changes in laboratory values				
All	4 (22.2)	3 (17.6)	5 (33.3)	1 (6.3)
Related to the study drug	3 (16.7)	1 (5.9)	3 (20.0)	1 (6.3)
Bleeding adverse events	2 (11.1)	2 (11.8)	2 (13.3)	1 (6.3)

Values are expressed as the number (%)

Abnormal changes in liver function test results were reported in one patient each in the prasugrel 2.5 mg, 5 mg, and 7.5 mg groups. In each case, the results normalized without treatment.

## Discussion

The IPA and PRI results of the present study show that prasugrel, in the range of 2.5–7.5 mg, has dose-dependent antiplatelet effects in Japanese patients with non-cardioembolic stroke. The results also suggest that the antiplatelet effects of prasugrel at these doses are more potent than those of clopidogrel 75 mg. In the clopidogrel group, unlike the prasugrel group, some patients had IPA < 10% at the end of treatment, indicating clopidogrel resistance. Patients who present with high on-treatment platelet reactivity are at significantly higher risk of recurrent cerebrovascular ischemic events (relative risk, 1.81), according to a recent systematic review and meta-analysis [18]. Therefore, this factor should be considered in the treatment and secondary prevention of stroke.

The low IPA in the clopidogrel group may be related to the presence in the group of patients with CYP2C19 polymorphisms; all except one had the IM or PM phenotype. In IMs and PMs, production of the active metabolite of clopidogrel is limited and therefore its antiplatelet effects are reduced. The wider range of IPA values for IMs in the clopidogrel group compared with IMs in the prasugrel groups suggests inconsistent inhibition of ADP-induced aggregation by clopidogrel. The US package insert for clopidogrel states that its effects may be reduced in patients with CYP2C19 polymorphisms. Furthermore, the results of a large-scale systematic review and meta-analysis have shown that, in patients with ischemic stroke or transient ischemic attack treated with clopidogrel, carriers of genetic polymorphisms, especially variants of *CYP2C19*, are at higher risk of stroke and vascular events than non-carriers [19].

Both prasugrel and clopidogrel are inactive prodrugs, and their active metabolites, which have similar chemical structures, have similar antiplatelet activity [20]. In the present study, the plasma concentration of the active metabolite was higher in all prasugrel groups than in the clopidogrel group. Higher exposure to active metabolite is associated with extensive platelet inhibition [20, 21]. Therefore, we attribute the higher IPA in patients treated with prasugrel to more efficient generation of its active metabolite.

Regarding safety, the incidence of AEs related to the study drug was higher in the prasugrel groups than in the clopidogrel group. However, there were no deaths, other serious AEs, or AEs resulting in discontinuation of the study drug. Bleeding AEs and abnormal changes in liver function test results, which are frequently reported adverse effects of thienopyridine antiplatelet agents, were recorded in a small number of patients. However, all cases improved without treatment. Therefore, no new safety concerns were identified in this study of treatment with prasugrel administered orally at 2.5–7.5 mg for 14 days.

The present study has several limitations. First, in the pharmacodynamic assessment, no statistical analyses were carried out to compare data for the prasugrel group with data for the clopidogrel group. Second, the small sample size means that the proportions of patients with the different CYP2C19 phenotypes may not reflect those in the real world. Third, it was not possible to identify CYP2C19 phenotype for all patients enrolled in the study.

## Conclusions

Prasugrel at daily doses of 2.5 mg, 5 mg, and 7.5 mg was well-tolerated and effective for inhibition of platelet aggregation in Japanese patients with non-cardioembolic stroke. The antiplatelet effects of prasugrel at all three doses were higher than those of clopidogrel 75 mg.

## Electronic supplementary material

Below is the link to the electronic supplementary material.

Supplemental Figure 1 Study design. Supplemental figure 2 Platelet reactivity index on day 14. ◊, Arithmetic mean; +, value within 1.5 to 3 quartiles of the box. Data from one patient in the clopidogrel group was excluded because of insufficient data. (PPTX 65 kb)

## References

[1] Joint Committee on Guidelines for the Management of Stroke (2015). Japanese guidelines for the management of stroke 2015.

[2] Cuisset T, Loosveld M, Morange PE, Quilici J, Moro PJ, Saut N, Gaborit B, Castelli C, Beguin S, Grosdidier C, Fourcade L, Bonnet JL, Alessi MC (2012). CYP2C19*2 and *17 alleles have a significant impact on platelet response and bleeding risk in patients treated with prasugrel after acute coronary syndrome. JACC Cardiovasc Interv.

[3] Frere C, Cuisset T, Morange PE, Quilici J, Camoin-Jau L, Saut N, Faille D, Lambert M, Juhan-Vague I, Bonnet JL, Alessi MC (2008). Effect of cytochrome p450 polymorphisms on platelet reactivity after treatment with clopidogrel in acute coronary syndrome. Am J Cardiol.

[4] Hulot JS, Bura A, Villard E, Azizi M, Remones V, Goyenvalle C, Aiach M, Lechat P, Gaussem P (2006). Cytochrome P450 2C19 loss-of-function polymorphism is a major determinant of clopidogrel responsiveness in healthy subjects. Blood.

[5] Mega JL, Close SL, Wiviott SD, Shen L, Hockett RD, Brandt JT, Walker JR, Antman EM, Macias W, Braunwald E, Sabatine MS (2009). Cytochrome p-450 polymorphisms and response to clopidogrel. N Engl J Med.

[6] Nagashima Z, Tsukahara K, Morita S, Endo T, Sugano T, Hibi K, Himeno H, Fukui K, Umemura S, Kimura K (2013). Platelet reactivity in the early and late phases of acute coronary syndromes according to cytochrome P450 2C19 phenotypes. J Cardiol.

[7] Paré G, Mehta SR, Yusuf S, Anand SS, Connolly SJ, Hirsh J, Simonsen K, Bhatt DL, Fox KA, Eikelboom JW (2010). Effects of CYP2C19 genotype on outcomes of clopidogrel treatment. N Engl J Med.

[8] Tang XF, Zhang JH, Wang J, Han YL, Xu B, Qiao SB, Wu YJ, Chen J, Wu Y, Chen JL, Gao RL, Yang YJ, Yuan JQ (2013). Effects of coexisting polymorphisms of CYP2C19 and P2Y_12_ on clopidogrel responsiveness and clinical outcome in patients with acute coronary syndromes undergoing stent-based coronary intervention. Chin Med J (Engl).

[9] Zhang L, Chen Y, Jin Y, Qu F, Li J, Ma C, Yang J, Xu B, Wang H, Li X, Li Y, Zhang Y, Lu C, Yin T (2013). Genetic determinants of high on-treatment platelet reactivity in clopidogrel treated Chinese patients. Thromb Res.

[10] Mega JL, Simon T, Collet JP, Anderson JL, Antman EM, Bliden K, Cannon CP, Danchin N, Giusti B, Gurbel P, Horne BD, Hulot JS, Kastrati A, Montalescot G, Neumann FJ (2010). Reduced-function CYP2C19 genotype and risk of adverse clinical outcomes among patients treated with clopidogrel predominantly for PCI: a meta-analysis. JAMA.

[11] Yamamoto K, Hokimoto S, Chitose T, Morita K, Ono T, Kaikita K, Tsujita K, Abe T, Deguchi M, Miyagawa H, Saruwatari J, Sumida H, Sugiyama S, Nakagawa K, Ogawa H (2011). Impact of CYP2C19 polymorphism on residual platelet reactivity in patients with coronary heart disease during antiplatelet therapy. J Cardiol.

[12] Liu R, Zhou ZY, Chen YB, Li JL, Yu WB, Chen XM, Zhao M, Zhao YQ, Cai YF, Jin J, Huang M (2016). Associations of CYP3A4, NR1I2, CYP2C19 and P2RY12 polymorphisms with clopidogrel resistance in Chinese patients with ischemic stroke. Acta Pharmacol Sin.

[13] Yokoi H, Kimura T, Isshiki T, Ogawa H, Ikeda Y (2012). Pharmacodynamic assessment of a novel P2Y12 receptor antagonist in Japanese patients with coronary artery disease undergoing elective percutaneous coronary intervention. Thromb Res.

[14] Brandt JT, Close SL, Iturria SJ, Payne CD, Farid NA, Ernest CS, Lachno DR, Salazar D, Winters KJ (2007). Common polymorphisms of CYP2C19 and CYP2C9 affect the pharmacokinetic and pharmacodynamic response to clopidogrel but not prasugrel. J Thromb Haemost.

[15] Wiviott SD, Braunwald E, McCabe CH, Montalescot G, Ruzyllo W, Gottlieb S, Neumann FJ, Ardissino D, De Servi S, Murphy SA, Riesmeyer J, Weerakkody G, Gibson CM, Antman EM, TRITON-TIMI 38 Investigators (2007). Prasugrel versus clopidogrel in patients with acute coronary syndromes. N Engl J Med.

[16] Umemura K, Ikeda Y, Kondo K (2016). Pharmacokinetics and pharmacodynamics of prasugrel in healthy Japanese subjects. Drug Metab Pharmacokinet.

[17] Yi X, Wang C, Liu P, Fu C, Lin J, Chen Y (2016). Antiplatelet drug resistance is associated with early neurological deterioration in acute minor ischemic stroke in the Chinese population. J Neurol.

[18] Fiolaki A, Katsanos AH, Kyritsis AP, Papadaki S, Kosmidou M, Moschonas IC, Tselepis AD, Giannopoulos S (2017). High on treatment platelet reactivity to aspirin and clopidogrel in ischemic stroke: a systematic review and meta-analysis. J Neurol Sci.

[19] Pan Y, Chen W, Xu Y, Yi X, Han Y, Yang Q, Li X, Huang L, Johnston SC, Zhao X, Liu L, Zhang Q, Wang G, Wang Y, Wang Y (2017). Genetic polymorphisms and clopidogrel efficacy for acute ischemic stroke or transient ischemic attack: a systematic review and meta-analysis. Circulation.

[20] Sugidachi A, Ogawa T, Kurihara A, Hagihara K, Jakubowski JA, Hashimoto M, Niitsu Y, Asai F (2007). The greater in vivo antiplatelet effects of prasugrel as compared to clopidogrel reflect more efficient generation of its active metabolite with similar antiplatelet activity to that of clopidogrel’s active metabolite. J Thromb Haemost.

[21] Small DS, Kothare P, Yuen E, Lachno DR, Li YG, Winters KJ, Farid NA, Ni L, Jakubowski JA, Salazar DE, Thieu VT, Payne CD (2010). The pharmacokinetics and pharmacodynamics of prasugrel in healthy Chinese, Japanese, and Korean subjects compared with healthy Caucasian subjects. Eur J Clin Pharmacol.

