# Supplementary material for: Pharmacodynamic assessment of prasugrel and clopidogrel in patients with non-cardioembolic stroke: a multicenter, randomized, active-control clinical trial
Source: J Thromb Thrombolysis. 2019 Oct 23;49(1):10–7. doi: 10.1007/s11239-019-01926-6 (PMC6954144; doi:10.1007/s11239-019-01926-6)
Supplement: Supplementary file 1 — Supplemental Figure 1 Study design. Supplemental figure 2 Platelet reactivity index on day 14. ◊, Arithmetic mean; +, value within 1.5 to 3 quartiles of the box. Data from one patient in the clopidogrel group was excluded because of insufficient data. (PPTX 65 kb) [file 11239_2019_1926_MOESM1_ESM.pptx]

## Slide 1
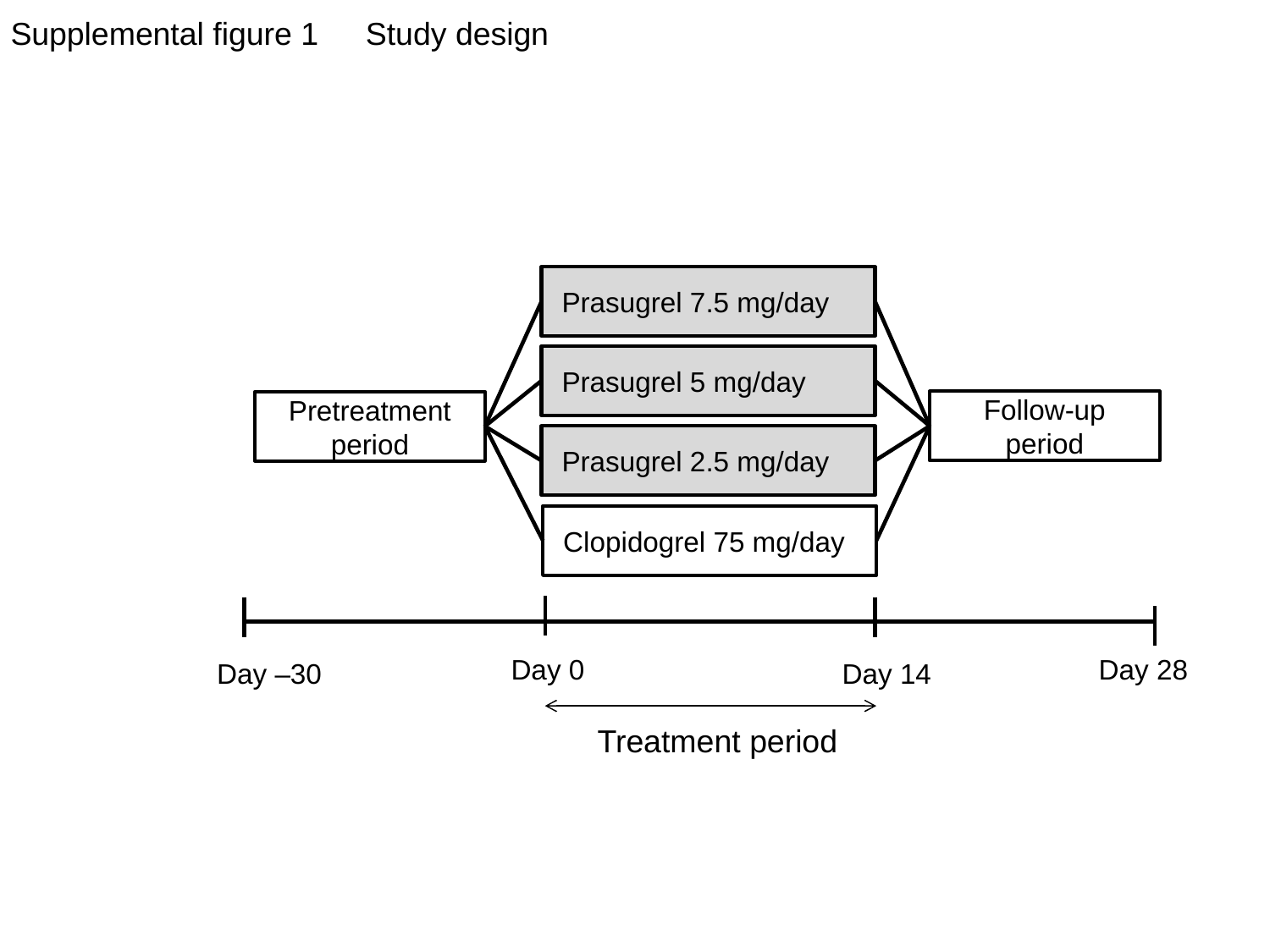

Supplemental figure 1　Study design
 Prasugrel 7.5 mg/day
 Prasugrel 5 mg/day
Follow-up
period
Pretreatment
period
 Prasugrel 2.5 mg/day
 Clopidogrel 75 mg/day
Day 0
Day 14
Day –30
Day 28
Treatment period

## Slide 2
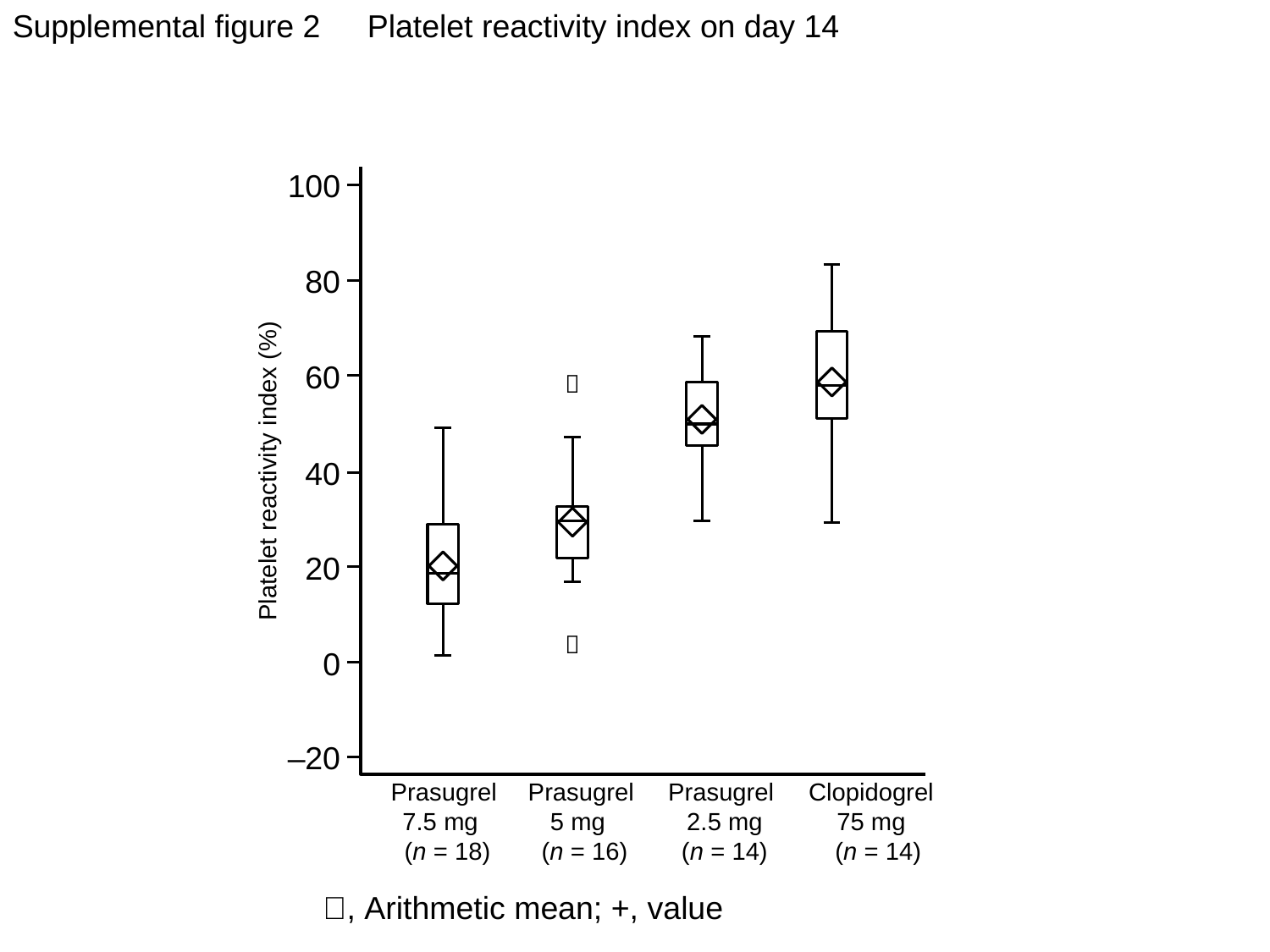

Supplemental figure 2　Platelet reactivity index on day 14
100
80
60
40
20
0
–20
＋
Platelet reactivity index (%)
＋
Prasugrel
7.5 mg
(n = 18)
Prasugrel
5 mg
(n = 16)
Prasugrel
2.5 mg
(n = 14)
Clopidogrel
75 mg
 (n = 14)
, Arithmetic mean; +, value
